# Pseudotumour Cerebri Syndrome in China: A Cohort Study

**DOI:** 10.1038/s41598-020-58080-w

**Published:** 2020-01-27

**Authors:** Qian Chen, Chaoyi Feng, Guixian Zhao, Weimin Chen, Min Wang, Xinghuai Sun, Yan Sha, Zhenxin Li, Guohong Tian

**Affiliations:** 1 Department of Ophthalmology, Eye Ear Nose and Throat Hospital of Fudan University, Shanghai, China; 2 Department of Neurology, Huashan Hospital, Fudan University, Shanghai, China; 3 Department of Neurology, Deji Hospital, Shanghai, China; 4 Department of Radiology, Eye Ear Nose and Throat Hospital of Fudan University, Shanghai, China; 5 NHC Key Laboratory of Myopia (Fudan University), Key Laboratory of Visual Impairment and Restoration, Shanghai, China

**Keywords:** Optic nerve diseases, Hydrocephalus

## Abstract

Pseudotumour cerebri syndrome (PTCS) remains to be fully investigated in Chinese patients and our study reported PTCS-related clinical differences between Chinese patients and Western patients. This study enrolled 55 consecutive patients (females: 44, median age: 37 y, age range: 14–62 y) with PTCS diagnosed from October 2015 to December 2017. Nine (16.4%, females) patients had primary PTCS, and 46 (83.6%) had secondary PTCS (*P* = 0.001). At presentation, 81.8% of patients had grade >3 papilloedema, with 23.6% having diffusely constricted fields. Mean subarachnoid space around the optic nerve measured by retrobulbar ultrasonography during lumbar puncture was 1.12 ± 0.17 mm and decreased to 0.86 ± 0.11 mm after treatment. Optical coherence tomography (OCT) showed that 92.9% of eyes with intact macular ganglion cell-inner plexiform layer (GCIPL) at baseline had good outcomes after treatment. Patients’ demographic and clinical characteristics showed that secondary PTCS was more common than primary idiopathic intracranial hypertension in Chinese patients. Polycystic ovarian syndrome was the main associated factor in females. Poor visual function was common at presentation. Noninvasive ocular ultrasonography and OCT are the prognostic indicators of PTCS treatment in intracranial pressure and visual function, respectively, after PTCS treatment.

## Introduction

Pseudotumour cerebri syndrome (PTCS), proposed by Friedman *et al*.^1^ in 2013 describes increased intracranial pressure (ICP) of unknown cause. PTCS can be primary or secondary based on its aetiology. The term idiopathic intracranial hypertension (IIH) is assigned to patients with primary PTCS, and is predominant in women of childbearing age with obesity^2–4^.

The global incidence of IIH is 12~20 per 100 000 people per year among women of childbearing age with obesity and 0.5~2 per 100 000 people in the general population^5–7^, while the reported rate in Japan is only 0.03 per 100 000 people per year^8^. A 28-year retrospective review of data at a tertiary hospital on Chinese patients with IIH reports that only 12 patients with IIH were evaluated, and obesity might not be an associated risk factor in Asian patients^9,10^. A recent meta-analysis reported correlation between IIH incidence and country-specific obesity prevalence^11^.

PTCS or IIH is not fully understood by ophthalmologists and neurologists in Mainland China. In this study, the term PTCS is used because of its atypical presentation (male, non-obese, associated medical conditions) in many Chinese patients. Our previously published work, and many other related studies show high sensitivity of noninvasive high resolution (20 Hz) transbulbar ultrasonography in predicting increased ICP in patients with bilateral disc oedema^12–16^, and hence ultrasonography was included in this study. Optical coherence tomography (OCT) is used routinely in neuro-ophthalmological evaluations^17–20^, and we employed it to grade papilloedema and measured the peripapillary retinal nerve fibre layer (RNFL) and macular ganglion cell-inner plexiform layer (GCIPL) thicknesses at baseline and 1-year after treatment.

This study aimed to evaluate a cohort of consecutive Chinese patients with PTCS diagnosed at a tertiary ophthalmology hospital in Shanghai during a 2-year period who presented with various visual symptoms. The goals included both analysing patients’ demographic and clinical characteristics, and finding prognostic indicators of treatment.

## Methods

### Patient selection

This retrospective cohort study enrolled consecutive patients with PTCS diagnosed at the neuro-ophthalmology division of the Eye, Ears, Nose, and Throat Hospital of Shanghai, China enrolled from October 2015 to December 2017. This study complied with the tenets of the Declaration of Helsinki. Written signed consent forms for study participation were obtained through a study protocol approved by the hospitals’ ethics committee. All study participants provided written informed consent, and the consent form was signed by a guardian for participants younger than 18 years of age.

The inclusion criteria for diagnosing PTCS according to Friedman *et al*.^7^ were as follows: (1) optic disc oedema confirmed on ophthalmoscopy, including secondary atrophy after disc oedema; (2) normal neurological examination except for cranial nerve VI abnormalities; (3) brain magnetic resonance imaging showing normal brain parenchyma without evidence of hydrocephalus, mass, or structural lesions; (4) lumbar puncture cerebrospinal fluid open pressure measurement of ≥250 mmH_2_O in adults and ≥280 mmH_2_O in children; and (5) normal cerebrospinal fluid analysis results. We excluded patients with contraindications for lumbar puncture, and those who were unwilling to undergo visual tests or complete a 1-year follow-up.

### Demographic data and ophthalmic examinations

We recorded the age, sex, BMI, and medical history such as recent weight gain, anaemia (haemoglobin < 12 g/dL), systemic hypertension, polycystic ovarian syndrome, sleep apnoea, renal failure, and drug use. We also recorded the presenting symptoms and the total duration of symptoms, for all patients.

The papilloedema seen on fundus examination was graded 0–5 according to the modified Frisén scale^1^, with grade 6 assigned to late-stage optic atrophy. The best-corrected visual acuity (BCVA) was measured using the revised standard Snellen chart in China and grouped into five grades as follows: ≥0.8 (≥20/25); 0.4–0.7 (20/50–20/30); 0.1–0.3 (20/200–20/60); < 0.1 to >counting fingers; and counting fingers to no light perception. Visual fields (static or kinetic) were evaluated if the BCVA was better than 0.1 and graded on a scale from 1–5 according to Szewka *et al*.^17^ with some modifications as: (1) normal, (2) enlargement of the blind spot, (3) nasal or temporal defect, (4) arcuate defect, and (5) diffusely constricted.

### Ocular transbulbar ultrasonography

Patients underwent 20-MHz high-resolution transbulbar ultrasonography (Aviso, Quantel Medical, Clermont-Ferrand, France) by an experienced ultrasound technician. Using ultrasonography, we measured the SAS of the optic nerve, 3 mm behind the lamina cribrosa and confirmed the presence of drusen and scleritis for exclusion, according to our previous study^7^.

### Optical coherence tomography

OCT imaging was performed with the Cirrus 5000 (Cirrus HD-OCT; Carl Zeiss Meditec AG, Jena, Germany). Peripapillary RNFL thickness measurements were obtained using the machine’s Optic Disc 200 × 200 protocol that evaluates 3.4-mm-diameter circles around the optic disc. The macular GCIPL measurements were obtained using the macular cube 512 A-scan × 128 B-scan protocol over a 6 × 6-mm^2^ area centred on the fovea. We used the in-built analysis software to evaluate the mean and minimum thickness of the combined GCIPL measurements, including measurements at a signal strength of ≥7.

### Statistics

Patients’ demographic characteristics are presented as means and percentages and compared by analysis of variance or chi-squared test. *P* < 0.05 was considered statistically significant. We used the rank sum test to evaluate the grade of papilloedema, visual acuity, and visual field tests. All analyses were performed using IBM SPSS statistics for windows, version 19.0 (IBM Corp., Armonk, NY).

### Ethics approval and informed consent

The Institutional Ethics Review Board of the Eye Ear Nose and Throat Hospital of the Fudan University Shanghai approved the study protocol, and written informed consent was obtained from all participants (No. KJ-2011-04). The methods were carried out in accordance with the relevant guidelines and regulations. All study participants provided written informed consent, and the consent form was signed by a guardian for participants younger than 18 years.

## Results

### Demographics and medical conditions

A total of 55 patients (110 eyes) with PTCS were evaluated. Nine (16.36%) patients with unknown underlying aetiology were diagnosed with primary PTCS, and the remaining 46 (83.64%) patients with underlying causes were diagnosed with secondary PTCS (*P* = 0.001). Demographic characteristics of the patients are shown in Table 1. The mean ages of patients with primary PTCS vs. secondary PTCS were 23.78 ± 6.9 years vs. 40.63 ± 9.6 years, respectively (*P* = 0.001). All patients with primary PTCS and 76.08% with secondary PTCS were women (*P* = 0.001). The mean body mass index (BMI) of patients with primary and secondary PTCS was 29.43 ± 1.43 and 25.57 ± 0.40, respectively (*P* = 0.04).

**Table 1:** Demographic and clinical characteristics of patients with pseudotumor cerebri syndrome.

	Primary PTCS (*n* = 9, eye = 18)	Secondary PTCS (*n* = 46, eye = 92)	*P* value
Age, yrs (mean ± SD)	23.78 ± 6.9	40.63 ± 9.6	*P* = 0.001
Female (%)	9 (100%)	35 (76.08%)	*P* = 0.001
Race (Asian %)	100%	100%	
BMI (mean ± SD)	29.43 ± 1.43	25.57 ± 0.40	*P* *=* 0.04
Contributing medications			
Anemia (n, %)	0	22 (47.82%)	
Polycystic ovarian (n, %)	0	33 (71.74%)	
Hypertension (n, %)	0	12 (26.09%)	
Sleep apnea (n, %)	0	11 (23.91%)	
Renal failure (n, %)	0	1 (2.17%)	
Steroids taking (n, %)	5 (55.56%)	26 (56.52%)	
Duration of symptoms, (months)	27.06 ± 18.98	32.29 ± 19.23	*P* *=* 0.05
Presenting symptoms			
Headache (n, %)	3 (33.33%)	7 (15.22%)	
TVO (n, %)	6 (66.67%)	25 (54.35%)	*P* *=* 0.05
Pulsatile tinnitus (n, %)	1 (11.11%)	12 (26.09%)	
Diplopia (n, %)	2 (22.22%)	2 (4.35%)	
Visual decrease (n, %)	7 (77.78%)	43 (93.48%)	*P* *=* 0.02
LP pressure (mm H_2_O)	325.56 ± 12.59	328.48 ± 6.01	

PTCS = pseudotumor cerebri; BMI = body mass index; TVO = transient visual obscurations; LP = lumbar puncture

The medical conditions associated with secondary PTCS included: polycystic ovarian syndrome in 33 (71.74%), anaemia in 22 (47.82%), hypertension in 12 (26.09%), and severe sleep apnoea in 11 (23.91%, male) patients. Only one patient (a 62-year-old man) had chronic renal dysfunction and anaemia in the secondary PTCS group. Steroids were used by 55.56% and 56.52% patients with primary and secondary PTCS, respectively (*P* = 0.70).

### Ophthalmic characteristics

The durations of symptoms before diagnosis of primary and secondary PTCS were 27.06 ± 18.98 and 32.29 ± 19.23 months, respectively (*P* = 0.05). The most common presenting symptom was patient-reported decreased vision (primary PTCS: 77.78%; secondary PTCS: 93.48%; *P* = 0.02), and the second most common symptom was transient visual obscuration (primary PTCS: 66.67%; secondary PTCS: 54.35%, *P* = 0.05). Other presenting symptoms included headache, pulsatile tinnitus, and diplopia (Table 1).

Grade 2–3 and grade 4–5 papilloedema at baseline by Frisén scale were seen most commonly in the primary and secondary PTCS groups, respectively (Table 2). Late-stage optic atrophy (grade 6) occurred in only one (5.56%) patient in the primary PTCS group and in 17 (18.48%) patients in the secondary PTCS group (*P* = 0.01). The baseline visual acuity was >20/25 in 88.89% and 63.04% patients with primary and secondary PTCS, respectively (*P* = 0.04). Very poor visual acuity occurred only with late-stage optic atrophy in the secondary PTCS group; 9.78% patients had visual acuity worse than 20/200 to > counting fingers, and 3.26% patients had visual acuity of counting fingers to no light perception.

**Table 2:** Frisén scale of papilloedema, visual acuity, and visual field results in patients with pseudotumor cerebri (baseline).

	Primary PTCS (*n* = 9, eye = 18)	Secondary PTCS (*n* = 46, eye = 92)	*P* value
**Papilloedema grade (eyes, %)**			**P** > **0.05**
Grade 0–1	4 (22.22%)	5 (5.43%)	P > 0.05
Grade 2–3	8 (44.44%)	21 (22.83%)	P > 0.05
Grade 4–5	5 (27.78%)	49 (53.26%)	P > 0.05
Grade 6	1 (5.56%)	17 (18.48%)	*P* *=* 0.01
**Visual acuity (eyes, %)**			***P*** = **0.04**
≥20/25	16 (88.89%)	58 (63.04%)	
20/50–20/30	1 (5.56%)	15 (16.30%)	
20/200–20/60	1 (5.56%)	7 (7.61%)	
<20/200->CF	0	9 (9.78%)	
CF-NLP	0	3 (3.26%)	
**Visual field**			P = 0.04
Normal	1 (5.56%)	5 (5.44%)	
Enlargement of the blind spot	16 (88.89%)	37 (40.22%)	
Nasal or temporal defect	0	14 (15.22%)	
Arcuate defect	0	9 (9.78%)	
Diffusely constricted	1 (5.56%)	27 (29.35%)	

PTCS = pseudotumor cerebri syndrome; CF = counting fingers; NLP = no light perception. *P* = statistically significant difference for group comparisons.

The patients’ visual field defects at baseline were classified into separate grades for evaluation. The most common type of visual field defect in both groups was the enlargement of the blind spot (primary PTCS: 88.89%, secondary PTCS: 40.22%). Diffusely constricted visual field was present in 29.35% patients with secondary PTCS compared to 5.56% patients with primary PTCS (*P* = 0.04).

### Transbulbar ultrasonography

The mean subarachnoid space (SAS) measured at baseline was 1.01 ± 0.17 and 1.14 ± 0.13 mm in the primary and secondary PTCS groups, respectively (*P* = 0.07). At 1-year post treatment, the mean SAS decreased to 0.89 ± 0.08 mm (*P* = 0.04) and 0.98 ± 0.12 mm (*P* = 0.04) in the primary and secondary PTCS groups, respectively.

### Optical coherence tomography

Peripapillary RNFL and macular GCIPL were measured. Reliable data were obtained from 18 eyes in the primary PTCS group and 60 eyes in the secondary PTCS group. The mean GCIPL thickness was 80.2 ± 8.9 and 60.5 ± 4.8 μm at baseline in the primary and secondary PTCS groups, respectively (*P* = 0.01). After treatment, at the 1 -year follow-up, 52/58 eyes with intact GCIPL at baseline had BCVA ≥ 20/25.

## Discussion

The major findings of this study are, (1) IIH seen in women with obesity was not common in our cohort of Chinese patients, and (2) PTCS from secondary causes such as polycystic ovarian syndrome, anaemia, sleep apnoea (in men), and hypertension was present in >80% of our patients with PTCS. All nine patients diagnosed with IIH were women, with a median age of 24 years and mean BMI of 29.4. According to the World Health Organization BMI scale^21,22^, only 2/9 of our patients will be classified as class I/II obese (BMI: 30–39.9), and none had a BMI > 36 in our cohort. Several small-sample studies and a meta-analysis report the differences in BMI between Asian and Western patients^8–10,23^. Although the actual incidence of IIH in Chinese patients remains unknown, the incidence is probably lower than in the Western population, since IIH appears to occur only in overweight Chinese women in their mid-twenties. The mean BMI was 25.6 in the secondary PTCS group, with most patients within the normal BMI range.

**Figure 1:**
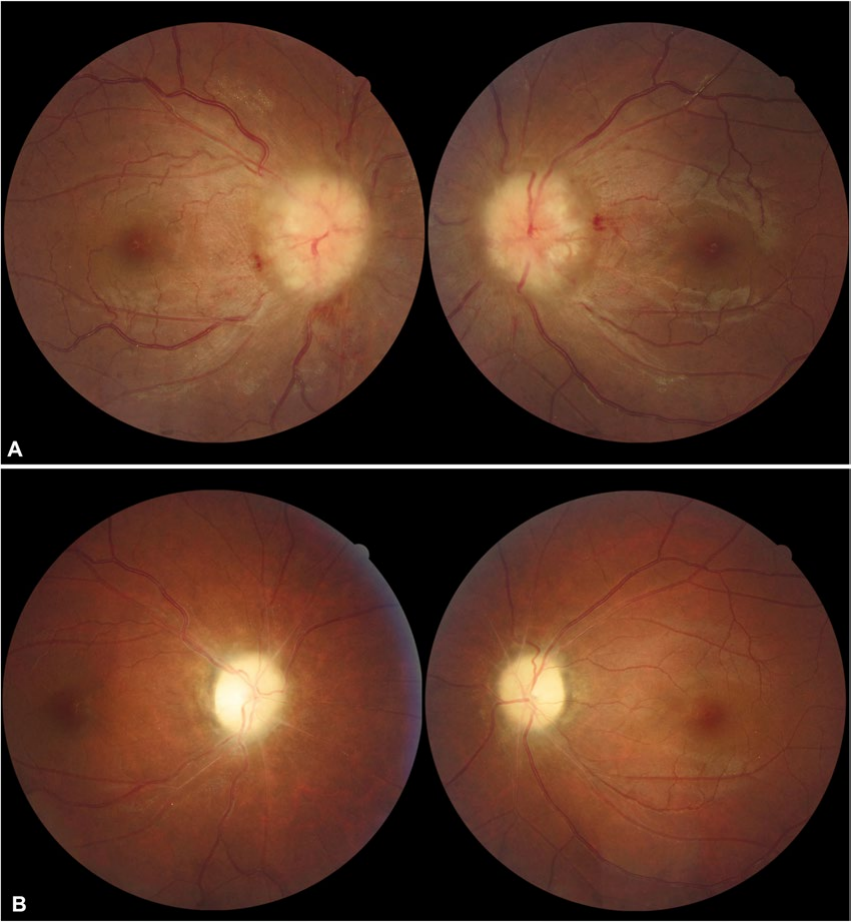
Fundus photographs of a 37-year-old patient with pseudotumour cerebri syndrome, polycystic ovarian syndrome, and anaemia with bilateral decrease in vision for 1 year. The visual acuity measured was 20/100 right eye and 20/25 left eye. (**A**) Optic disc was swollen with haemorrhages, and obscuration disc vessels (grade 5 papilloedema) at presentation. (**B**) After 6 months of methazolamide treatment and anaemia correction, the optic disc swelling resolved, and optic atrophy and vessel narrowing ensued with stabilization of visual acuity.

Polycystic ovarian syndrome and anaemia were the most common underlying comorbidities in our patients with secondary PTCS. The pathophysiologic link between polycystic ovarian syndrome and IIH is not fully understood, but may be related to several endocrine abnormalities^24–26^. A pertinent medical history for polycystic ovarian syndrome along with a gynaecological consultation appears to be important in female patients with suspected PTCS. Anaemia, the second most common comorbidity in our female cohort, is usually caused by iron deficiency and is associated with polycystic ovarian syndrome. Our data shows that anaemia is a likely risk factor for papilloedema in female patients with PTCS without obesity. The underlying mechanism might be related to ischemia of the RNFL and increased blood vessel permeability, which worsens the exudate in the papilloedema (Fig. 1). When anaemia was corrected, optic disc oedema resolved quickly in some patients, which supports the hypothesis that anaemia is a risk factor for PTCS. Sleep apnoea is also as a known cause of PTCS^27,28^, and was found only in male patients in our study. Underdiagnosed male patients with sleep apnoea in Mainland China usually present with late-stage optic atrophy, rather than with papilloedema. A complete medical history with a recording of the fundus and visual field changes are useful in making a final diagnosis of PTCS. The percentages of patients taking steroids were similar in the primary and secondary PTCS groups, in our study. Steroids were prescribed for the wrongly diagnosed optic neuritis or disc vasculitis instead of papilloedema in many patients. In turn, the long-term use of steroids exacerbated obesity and papilloedema.

Presentation with decreased vision was high in the secondary PTCS group. Most patients with primary PTCS presented with grade 2–3 papilloedema, whereas those with secondary PTCS presented with grade 4–5 papilloedema. Nearly one-fifth of our patients already had optic atrophy with poor visual acuity. These findings indicated that late-stage patients with optic disc pallor rather than papilloedema present management challenges for ophthalmologists in China.

**Figure 2:**
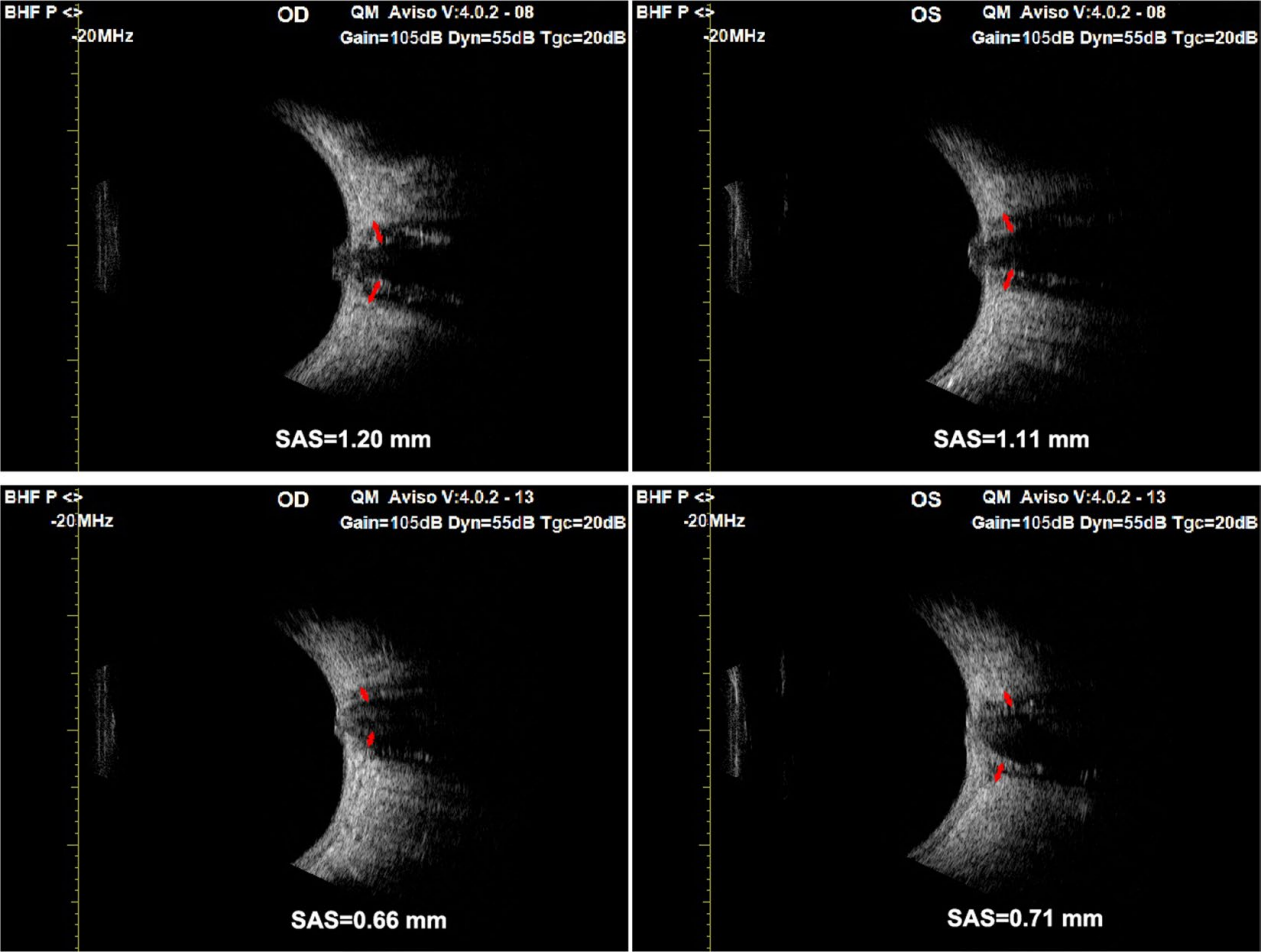
High resolution transbulbar ultrasonography of the subarachnoid space of optic nerve, 3 mm behind the lamina cribrosa. The subarachnoid space was 1.20 mm right eye and 1.11 mm left eye at presentation (top) with bilateral papilloedema grade 5 (the same patient as Fig. 1). After 6 months of treatment, the repeat scan measured the subarachnoid space at 0.66 mm right eye and 0.71 mm left eye (below) with resolution of optic disc swelling. SAS: subarachnoid space.

**Figure 3:**
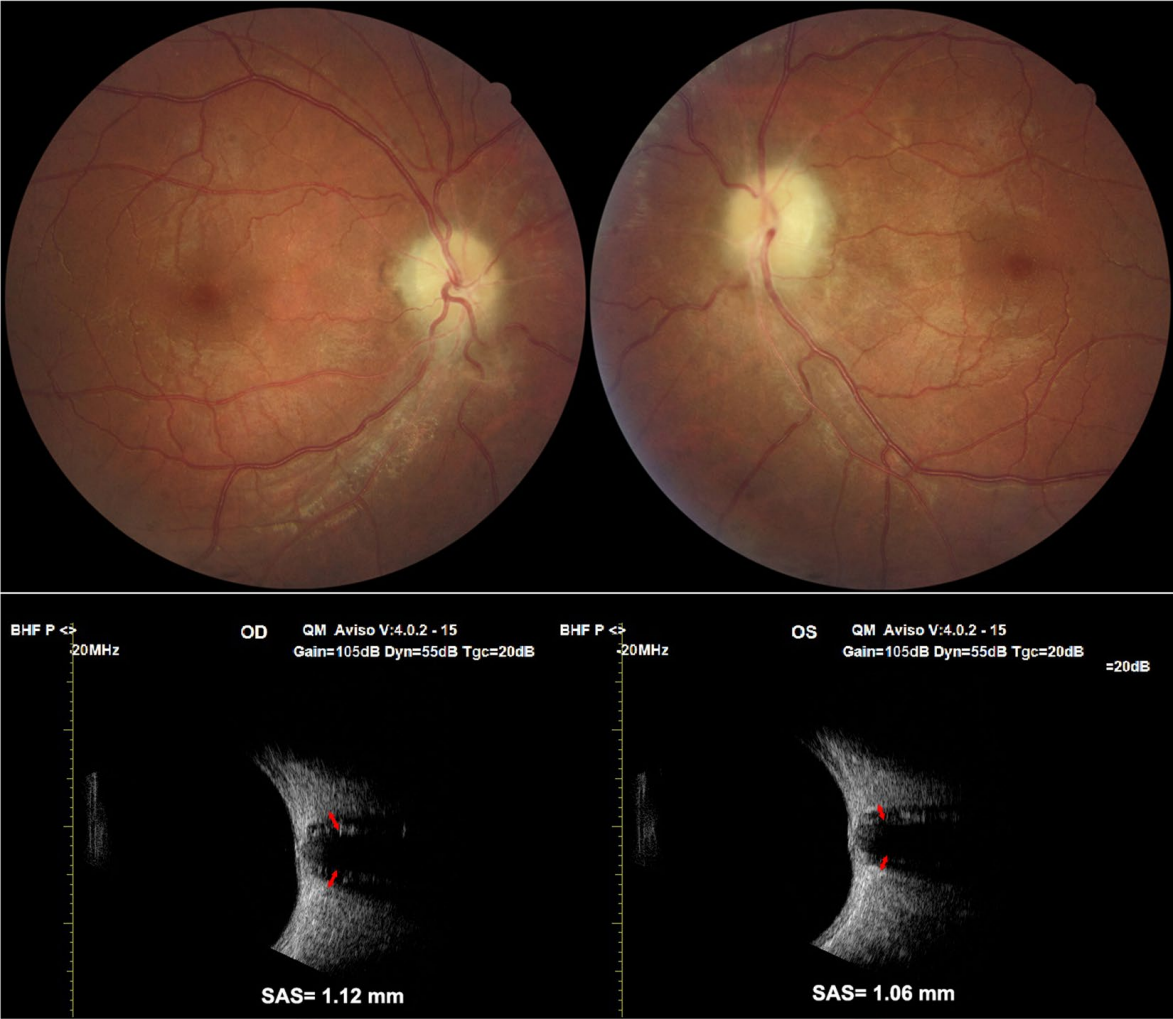
(**a**) Fundus photographs of a 36-year-old male with severe sleep apnoea who presented with bilateral decrease in vision for 2 years. The optic disc was swollen and pale with narrowed blood vessels. (**b**) The subarachnoid space measured was 1.12 mm right eye and 1.06 mm left eye at presentation. Lumbar puncture measured the ICP as 350 mmH_2_O. High resolution transbulbar ultrasonography predicating increased intracranial pressure (ICP) in a patient with late-stage pseudotumour and optic atrophy.

The enlargement of SAS surrounding the optic nerve, 3 mm behind the lamina cribrosa seen on noninvasive transbulbar ultrasonography was a great aid in differentiating papilloedema from bilateral disc oedema secondary to other causes^12^. A widened SAS of >1 mm strongly indicates a swollen disc secondary to increased ICP. In some of our patients with late-stage optic atrophy and PTCS, the SAS remained enlarged whereas the peripapillary RNFL atrophied (Figs. 2 and 3). A linear relationship between ICP and the size of the SAS remains undefined; however, the size of the SAS decreased in all of our successfully treated patients when the ICP decreased to < 200 mmH_2_O.

**Figure 4:**
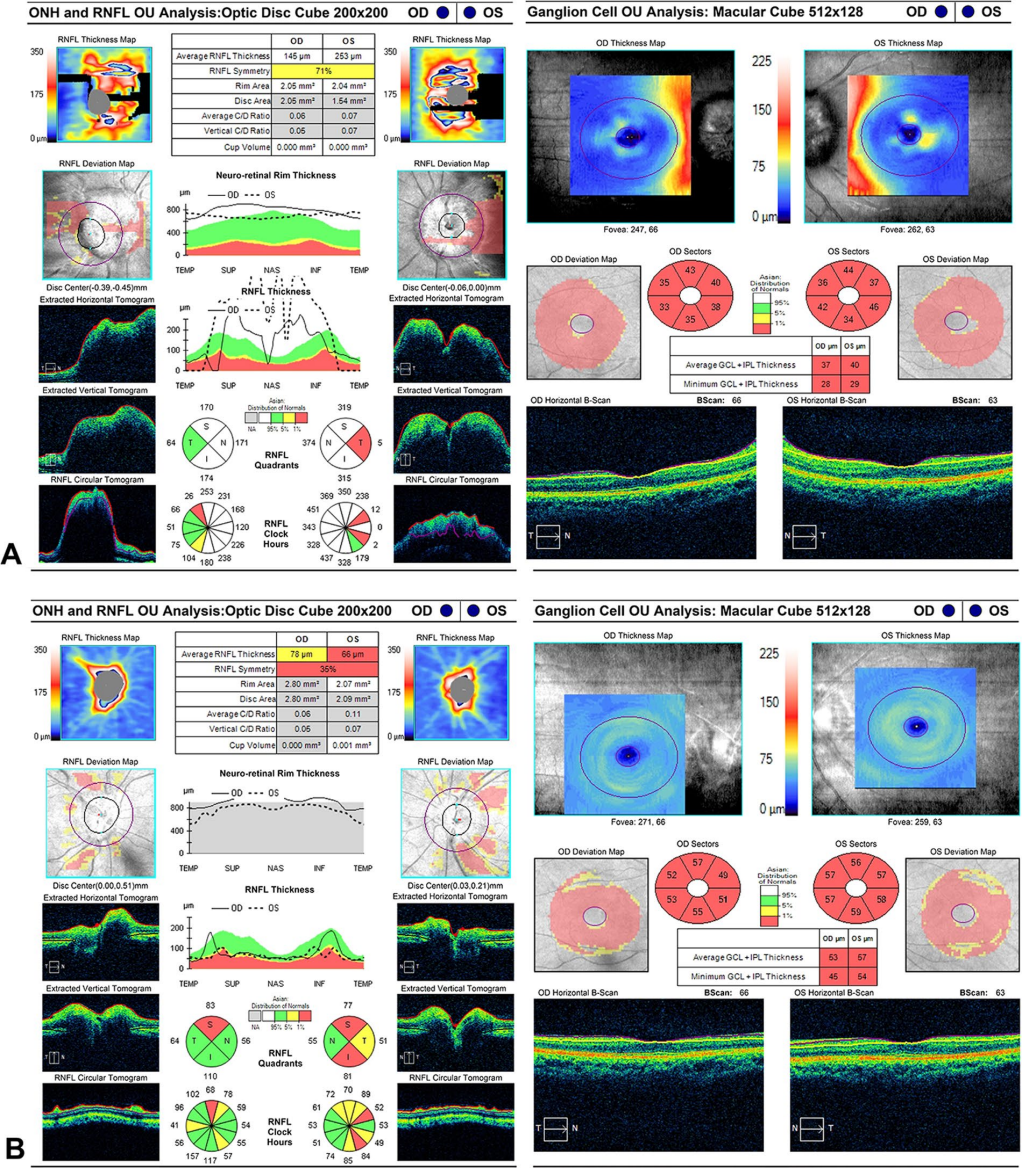
Optical coherence tomography (OCT) for evaluating pseudotumour cerebri syndrome. (**A**) Cirrus segmentation of the peripapillary RNFL shows artefact and unreliable parameters in severe grade 5 papilloedema, whereas macular GCIPL shows obviously thinning (the same patient in Fig. 1). (**B**) OCT repeated after 6 month of treatment in the same patient showed resolution of RNFL thinning but persistent GCIPL thinning, consistent with the poor visual outcome. GCIPL: ganglion cell-inner plexiform layer, RNFL: retinal nerve fibre layer. There were artifacts in segmentation of GCIPL thickness caused by swollen optic disc, the thickness measurement was accurate after therapy.

OCT is a revolutionary and widely used technique in neuro-ophthalmology^17,29^. Our data resonates with Chen *et al*. in that macular GCIPL thickness provides more information on papilloedema grade and prognosis than peripapillary RNFL thickness alone^30^. In our study, a preserved GCIPL compared with baseline indicated good visual function if the patient was treated successfully, whereas severe GCIPL thinning predicated a poor outcome with irreversible optic neuropathy (Fig. 4). However, OCT data becomes unreliable due to artefacts in patients with severe papilloedema, macular exudate, and subretinal fluid.

Methazolamide, originally used to treat glaucoma, is the only medication available in Mainland China to decrease ICP. In our experience, methazolamide (25 mg) is effective at 50–100 mg daily. Weight loss was encouraged for patients with BMI > 25^31^. Patients with anaemia were advised to consult their family physicians. Surgical interventions such as optic nerve sheath fenestration is rarely performed by ophthalmologists in China, and only one patient with intractable loss of visual function, secondary PTCS and methazolamide allergy was referred for venous sinus stenting^32^.

Our study has several limitations. Primarily, our sample size is small, especially the number of patients in the early stage of secondary PTCS. Other limitations include the retrospective study design and the lack of a defined treatment protocol. Moreover, the present study was conducted in a tertiary ophthalmology hospital in Shanghai, and the results obtained herein might not reflect the tendency in the general population.

In conclusion, PTCS is not widely recognised by ophthalmologists and neurologists in China, and as a result, some patients present with late-stage optic atrophy instead of papilloedema. Our study demonstrated that primary PTCS seen as IIH in obese individuals is rare in Chinese women, whereas secondary PTCS due to other medical conditions is more common. Our findings provide important insights into the roles of noninvasive ocular ultrasonography in predicting intracranial pressure and OCT in prognosing of visual function after PTCS treatment. Additionally, Further studies are needed to confirm these incidence data on Asian PTCS, to define prognostic indicators of PTCS treatment, and to evaluate treatments.

## Supplementary information

Supplementary Dataset 1.

## Data Availability

The datasets or analysed during the current study are available from the corresponding author on reasonable request.
